# Cotrimoxazole and targeted antibiotic prophylaxis for transrectal prostate biopsy: a single-center study

**DOI:** 10.1007/s00345-024-04969-4

**Published:** 2024-04-25

**Authors:** Matthias Jahnen, Thomas Amiel, Florian Kirchoff, Jacob W. Büchler, Kathleen Herkommer, Kathrin Rothe, Valentin H. Meissner, Jürgen E. Gschwend, Lukas Lunger

**Affiliations:** 1 Department of Urology, Klinikum Rechts der Isar, School of Medicine and Health, Technical University of Munich, Ismaninger Str. 22, 81675 Munich, Germany; 2 Institute for Medical Microbiology, Immunology and Hygiene, University Hospital Rechts der Isar, School of Medicine, Technical University of Munich, Munich, Germany

**Keywords:** Prostate biopsy, Antibiotic prophylaxis, Cotrimoxazole

## Abstract

**Purpose:**

The recent restriction on the use of fluoroquinolones for prophylaxis by the European Commission has left a gap in clear recommendations for practical antibiotic prophylaxis (PAP) for transrectal prostate biopsy (TRPB). This analysis investigated the viability of cotrimoxazole for PAP in TRPB.

**Methods:**

This analysis included *n* = 697 patients who underwent TRPB for suspected prostate cancer (PCa). All patients received either empiric PAP with four doses of cotrimoxazole 960 mg or targeted antibiotic prophylaxis in case of a positive rectal or urine screening for multiresistant gram-negatives. Infectious complications after TRPB, microbiological findings, and clinical characteristics were evaluated. A multivariable logistic regression model was calculated to identify variables associated with infectious complications.

**Results:**

Of the cohort, 86% (600/697) received PAP with cotrimoxazole, 1% (8/697) received cotrimoxazole plus an additional antibiotic, 4% (28/697) received amoxicillin + clavulanic acid, 4% (28/697) received fluoroquinolones, and 5% (33/697) received a single shot intravenous antibiotic prophylaxis with meropenem or piperacillin + tazobactam due to multiresistant microbiological findings in either pre-interventional urine culture or rectal swab. Infectious complications occurred in 2.6% (18/697) of patients. Fever was noted in 89% (16/18) of cases. Inpatient treatment was given to 67% (12/18) of affected patients, with 38% (7/18) having positive blood cultures, identifying cotrimoxazole-resistant *E. coli* strains in six out of seven cases. Multivariable logistic regression analysis revealed no clinically significant variables, including PAP with cotrimoxazole, as independent risk factors for an infectious complication.

**Conclusions:**

Using cotrimoxazole as PAP for TRPB in cases without multiresistant gram-negatives in pre-interventional urine cultures or rectal swabs seems feasible and practical.

## Introduction

Prostate biopsy is a regularly performed diagnostic procedure in men with suspected prostate cancer (PCa). Due to a slightly higher incidence of infectious complications following transrectal prostate biopsy (TRPB), perineal prostate biopsy has become the recommended procedure in recent years [1]. Population-based studies and meta-analyses indicate that transrectal and transperineal biopsies result in infectious complications in 2.8–7.6% and 0.5–3% of patients, respectively [2, 3]. In response to these findings, many urologists have transitioned to perineal biopsy, although TRPB remains an option when perineal biopsy is not feasible [1]. Due to the risk of introducing rectal bacteria into tissue or the bloodstream during TRPB, peri-interventional antibiotic prophylaxis (PAP) is strongly advised [1, 4]. However, the recent restriction on the use of fluoroquinolones for prophylaxis by the European Commission and the German Federal Institute for Drugs and Medical Devices has left a gap in clear recommendations for practical antibiotic prophylaxis for TRPB [5]. Previous studies have suggested one to two doses of oral fosfomycin 3g as suitable antibiotic prophylaxis [6–8]. However, regulatory constraints have limited the use of fosfomycin for this purpose [1]. Other alternatives, such as cephalosporins and aminoglycosides or the combination of multiple antibiotic agents, have proven effective [6]. Nonetheless, concerns about the widespread use of numerous broad-spectrum antibiotics, especially cephalosporins, contributing to antibiotic resistance, persist.

Current guidelines advocate for a rectal swab to detect multiresistant gram-negative bacteria [9] in the rectal flora and a urine culture to identify (multiresistant) bacteriuria before the biopsy, with targeted antibiotics recommended for suspicious results [1, 6, 10]. Still, these diagnostic measures often yield inconclusive results, necessitating calculated PAP in most patients.

Amid this ambiguity, we conducted a thorough analysis of local resistance patterns and established an approach utilizing cotrimoxazole as empirical PAP for TRPB [11]. Cotrimoxazole—a combination of trimethoprim and sulfamethoxazole—has activity against a broad spectrum of gram-positive and especially gram-negative bacteria, making it a suitable antibiotic agent for most genitourinary infections [12].

In this retrospective analysis of 697 biopsies, we aimed to illustrate the feasibility of an empiric PAP for TRPB with cotrimoxazole in cases where rectal swabs and urine cultures yielded inconspicuous results while evaluating the corresponding rates of infectious complications.

## Subjects and methods

### Design and procedure

This retrospective analysis included *n* = 697 patients who underwent TRPB for suspected PCa between November 2019 and January 2023 at the Department of Urology, Klinikum rechts der Isar, Technical University of Munich. The retrospective evaluation of the patient’s data was approved by the Ethics Committee of the Technical University of Munich (2022-151-S-KH; 2023-74-S-KH).

### Microbiology testing and antibiotic strategy

Before the biopsy, all men underwent a rectal swab, and a urine culture was obtained. In instances where no multiresistant gram-negatives according to German classification [9] were detected in the rectal swab and no urinary tract infection was identified, all patients received PAP with cotrimoxazole 960 mg. The four doses were administered the night before the biopsy, on the morning of the biopsy day, the evening following the biopsy, and the subsequent morning. In the event of detection of multiresistant gram-negatives in the rectal swab, antibiotic prophylaxis was adjusted according to the susceptibility testing, entailing a single shot of meropenem 1 g or piperacillin + tazobactam 4 g/0.5 g. In cases of urinary tract infections, patients were treated based on susceptibility testing for a peri-interventional duration of 3–5 days.

### Measures

#### Sociodemographic and clinical characteristics

The following sociodemographic and clinical data were obtained for this analysis: age at biopsy, PSA level at bx (ng/ml), prostate volume (ml), prior bx (yes; no), number of total cores taken, and detection of PCa (yes; no).

#### Microbiological and antibiotic characteristics

The following microbiological characteristics were considered for this analysis: results of pre-biopsy rectal swab, findings from pre-biopsy urine culture, the type of PAP administered, results of urine culture at readmission, and blood culture results upon readmission for infection.

#### Complications

Complications were assessed as readmissions for a follow-up time of 21 days after TRPB. Either of the following criteria was defined as an infectious complication (yes/no): fever, dysuria, and severe prostate pain.

Complications were further assessed regarding the presence of fever (yes/no), inpatient treatment (yes/no), positive blood culture results (yes/no), and admission to intensive care (yes/no).

### Statistical analysis

Descriptive statistics were calculated for all study variables. Variables with normal distribution are reported as mean and standard deviation (SD). Nonparametric data are reported as median and interquartile range (IQR) or range.

Univariable logistic regression analyses and a multivariable logistic regression model were calculated to identify variables (age, prostate volume, biopsy cores taken, and type of antibiotic prophylaxis (cotrimoxazole vs. targeted prophylaxis)) associated with an infectious complication (yes/no). Statistical significance was set at *p* < 0.05. All analyses were performed using SPSS (Version 26, IBM, Armonk USA). Graphs were created using Microsoft Office (Version 16.66.1, Microsoft, Redmond USA).

## Results

### Patient characteristics and bx results

A total of 697 men (age (SD): 65.9 (9.9) years) were evaluated for this retrospective study. All men received a TRPB with 14.7 (SD 3.4) biopsy cores extracted per biopsy. Further clinical characteristics can be found in Table 1.

**Table 1:** Sociodemographic and clinical characteristics of the study sample (*N* = 697)

Characteristic	
Mean (SD) age at biopsy (*years)* (*n* = 697; missing: 0)	65.9 (9.9)
Median (IQR) PSA (*ng/ml)* (*n* = 693; missing: 4)	7.2 (5.1)
Median (IQR) prostate volume *(ml)* (*n* = 610; missing: 87)	51.7(29.5)
Mean (SD) number of total cores extracted (*n* = 696; missing: 1)	14.7 (3.4)
No. patients with prostate cancer (%) (*n* = 697; missing: 0)	
Yes	496 (71.2)
No	201 (28.8)
No. of complications (%) (*n* = 697; missing: 0)	
No complications	664 (94.4)
Infectious complication	18 (2.6)
Urinary retention	5 (0.7)
Macrohematuria	5 (0.7)
Rectal bleeding	3 (0.4)
Post-intervention syncope	2 (0.3)

*No.* number, *PSA* prostate-specific antigen, *SD* standard deviation

**Fig. 1:**
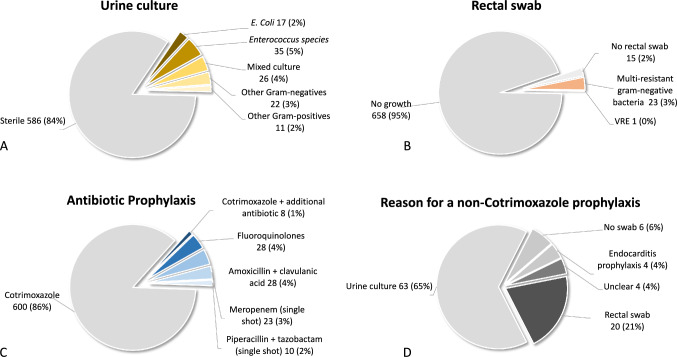
Pre-biopsy urine culture (**A**), rectal swab results (**B**), selected antibiotic prophylaxis (**C**), and reasons for non-cotrimoxazole antibiotic prophylaxis (**D**)

Of the total cohort, 86% (600/697) received empiric PAP with cotrimoxazole and 1% (8/697) received cotrimoxazole plus an additional antibiotic, while 4% (28/697) received amoxicillin + clavulanic acid, 4% (28/697) received fluoroquinolones and 5% (33/687) received a single shot intravenous antibiotic prophylaxis with either meropenem or piperacillin + tazobactam (Fig. 1C). Deviation from cotrimoxazole-based PAP primarily occurred when a positive urine culture identified uropathogens or a rectal swab identified multiresistant gram-negative bacteria (Fig. 1D). Commonly identified bacteria in urine cultures were *Enterococcus species* and *E. coli* (Fig. 1A). Multiresistant bacteria, according to German national classification, was detected in 3% (24%) of men via rectal swabs (Fig. 1B).

**Fig. 2:**
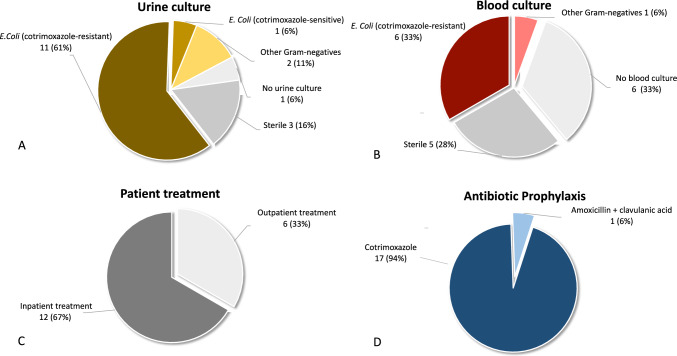
Urine culture (**A**) and blood culture (**B**) results of patients with post-biopsy infectious complications (2.6% (18/697)). Patient management inpatient vs. outpatient treatment (**C**) and choice of pre-biopsy antibiotic prophylaxis (**D**)

Infectious complications following TRPB occurred in 2.6% (18/697) of patients. Among these, 94% (17/18) had received an empiric PAP with cotrimoxazole (Fig. 2D). Fever was noted in 89% (16/18) of cases, while 11% (2/18) presented with dysuria without fever. 77% (14/18) of patients presented with a positive urine culture, identifying cotrimoxazole-resistant *E. coli* strains in 11 of 18 cases (Fig. 2A). Inpatient treatment was administered to 67% (12/18) of affected patients (Fig. 2C), with 39% (7/18) exhibiting positive blood cultures, identifying cotrimoxazole-resistant *E. coli* strains in six of seven cases (Fig. 2B). All patients experienced full recovery.

Multivariable logistic regression analysis revealed no clinically significant variables, including antibiotic prophylaxis with cotrimoxazole, as independent risk factors for an infectious complication (Table 2).

**Table 2:** Univariable and multivariable logistic regression to determine the association of selected clinical parameters with infectious complications

Clinical parameters	Univariable regression		Multivariable regression	
	OR (95% CI)	*P* value	OR (95% CI)	*P* value
Age (continuous^a^)	0.99 (0.95–1.04)	0.8	0.98 (0.94–1.04)	0.6
Prostate volume (continuous^a^)	1.00 (0.99–1.02)	0.9	1.00 (0.98–1.02)	0.9
Biopsy cores taken (continuous^a^)	0.96 (0.85–1.09)	0.5	0.99 (0.86–1.14)	0.9
Antibiotic prophylaxis (ref. targeted)				
Cotrimoxazole	1.24 (0.27–5.38)	0.8	1.50 (0.28–7.97)	0.6

*OR* odds ratio, *PSA* prostate-specific antigen, *ref.* reference, *95% CI* 95% confidence interval

^a^In steps of 1.0

## Discussion

Despite recent recommendations to perform transperineal prostate biopsies, TRPB remains a valuable alternative whenever transperineal prostate biopsies are impractical [1]. To mitigate the risk of biopsy-related infections, PAP is widely recommended [1, 6]. However, recent restrictions on the use of fluoroquinolones and fosfomycin for prophylaxis have created a gap in explicit PAP for TRPB [1, 5]. The use of cotrimoxazole in the peri-biopsy setting, although highly effective against gram-negative bacteria with comparable resistance rates to fluoroquinolones, is currently uncommon and not endorsed by international guidelines [10, 11]. Nevertheless, our data demonstrate that cotrimoxazole is a safe oral antibiotic option for PAP of TRPB after exclusion of rectal colonization by multiresistant gram-negative bacteria. Under this approach, infectious complications were recorded in 2.6% of patients, a rate comparable to studies assessing fluoroquinolone- or cephalosporin-based PAP (2.8–7.6%) [6]. Severe infectious complications leading to hospital admission were documented in 1.7% of patients, with bacteraemia found in 1.0%, comparable to rates following fluoroquinolone-based (2.7%) and non-fluoroquinolone-based (1.9%) PAP [3, 6, 10, 11, 13, 14].

The primary risk associated with TRPB is the potential to induce tissue or bloodstream infections caused by gram-negative bacteria through microtrauma in the rectal mucosa. In this context, the detection of multiresistant gram-negative bacteria in rectal swabs poses a significant risk of multiresistant bacteraemia. Our data show that 3% of men exhibited multiresistant gram-negative bacteria in pre-biopsy rectal swab. These men received antibiotic prophylaxis with either a single shot of intravenous meropenem or piperacillin + tazobactam 15 min before the biopsy based on the susceptibility testing, and none experienced infectious post-interventional infectious complications. Pre-biopsy urine culture detected asymptomatic bacteriuria in 16% of men, with *Enterococcus species* and *E. coli* being the most common bacteria. These men received PAP for 3–5 days based on the urine culture, most often fluoroquinolones or amoxicillin + clavulanic acid. One patient in this group who received amoxicillin + clavulanic acid had an infectious complication without bacteremia. These results emphasize that targeted antibiotic prophylaxis, guided by rectal swab and urine culture, plays a crucial role in preventing serious infectious complications following rectal prostate biopsy. In scenarios without a positive rectal swab or urine culture, the likelihood of colonialization with multiresistant bacteria is low. Cotrimoxazole seems to be a feasible antibiotic choice in such instances, as multivariable regression analysis did not indicate an increased risk of an infectious complication following empirical PAP with cotrimoxazole.

However, a limitation of targeted antibiotic prophylaxis is that screening for bacterial strains with a single antibiotic resistance, e.g., to cotrimoxazole, is not cost-effective due to the large number of different bacterial colonies in the rectum. Routine screening, therefore, typically focuses on identifying multiresistant antibiotic strains, which does not rule out the cultivation of bacteria strains with a single antibiotic resistance [13, 14]. Upon analyzing cases of men readmitted with an infectious complication following prostate biopsies, our investigation revealed that two-thirds of these cases were cotrimoxazole-resistant *E. coli*, as identified by urine and blood culture results. These findings not only highlight the importance of rectal swabs prior to transrectal prostate biopsy. In addition, knowledge of local antimicrobial resistance levels is crucial for selecting the appropriate antibiotic prophylaxis in cases where no multiresistant gram-negatives are detected in the rectal swab.

It is essential to acknowledge the limitations of our analysis, primarily its retrospective design and the absence of a control group. Despite these limitations, our study of nearly 700 consecutive biopsies provides meaningful data for comparison with previous research. Also, complications were assessed by reviewing patient records for readmission to the same hospital where the biopsy was conducted. It is acknowledged that some individuals might have sought medical care at other healthcare facilities, which may have led to these complications being missed. Nevertheless, all patients received comprehensive guidance to promptly seek readmission at the biopsy site if they developed a fever or further complications, thereby increasing the likelihood of accurately capturing severe complications in our analysis.

In conclusion, our findings suggest that utilizing cotrimoxazole as an antibiotic prophylaxis for transrectal biopsies, particularly in cases of negative urine culture and rectal screening for multiresistant bacteria, is both feasible and practical. This approach may be safely recommended in situations where perineal biopsy is not available, contributing to reducing the use of fluocinolones and cephalosporins.

## Data Availability

Data are available for bona fide researchers who request it from the authors.
